# Based on Unmodified Aptamer-Gold Nanoparticles Colorimetric Detection of Dexamethasone in Food

**DOI:** 10.3390/bios12040242

**Published:** 2022-04-14

**Authors:** Yadi Qin, Hayilati Bubiajiaer, Jun Yao, Minwei Zhang

**Affiliations:** 1 School of Pharmacy, Xinjiang Medical University, Xinyi Road, Urumqi 830054, China; xydqyd@stu.xjmu.edu.cn (Y.Q.); 107602201301@stu.xjmu.edu.cn (H.B.); 2 College Life Science & Technology, Xinjiang University, Shengli Road, Urumqi 830046, China

**Keywords:** aptamer, colorimetric, AuNPs, dexamethasone

## Abstract

Residue and illegal addition of Dexamethasone (DEX) in food has received widespread attention over the past few decades. Long-term intake of DEX will have a strong endocrine-disrupting effect, and there is an urgent need to develop highly sensitive and rapid on-site detection methods. In this work, a colorimetric sensor based on an unmodified aptamer and gold nanoparticles (Au NPs) was designed to detect DEX in milk and glucosamine. Under optimized conditions, the absorbance ratio of Au NPs increased linearly with DEX concentration over the range of 10–350 nmol/mL (r^2^ = 0.997), with a limit of detection (LOD) of 0.5 nmol/mL, and the recoveries ranged from 93.6 to 117%. To explore the interaction mechanism between aptamer and DEX, molecular docking and molecular dynamics simulations were applied to probe intermolecular interactions and structures of the complex. The establishment of aptamer-based sensors effectively avoids the antibody screening response, with a cost-efficient, excellent selective and great potential in DEX determination.

## 1. Introduction

Dexamethasone (DEX) is a synthetic hormone that is commonly used as an immunological and anti-inflammatory drug [1]. Long-term excessive intake of DEX can disrupt the nervous, endocrine, and digestive systems in humans [2,3]. Current European Union legislation restricts the maximum residue limits (MRLs) in dairy and restricts the abuse of DEX [4,5]. The EU and China stipulate that the MRL of DEX in milk is 0.3 ug/kg, and Japan is 0.02 mg/kg. Due to the increasing popularity of dietary supplements [6], some unscrupulous manufacturers have started adding corticosteroids to their products to enhance their efficacy [7]. These products are marketed as ‘natural products’ and can be found both online and in stores. However, the use of these products has significant health risks [8,9]. As a consequence, the analysis of DEX residues and illegal additions within food is very important.

Traditional analytical methods for determining dexamethasone residues are based on liquid chromatography (HPLC) with mass spectrometry (MS) detection via thermal spray interface with positive filament ionization. The results of these analytical methods are accurate and reproducible [10]. Furthermore, enzyme-linked immunosorbent assay (ELISA) has been employed in DEX assay. However, the immunological techniques have a high false-positive rate, and instrumental analytical methods are expensive, require highly trained staff, and cannot be deployed in field settings [11,12]. As a result, there is still a need to create effective and sensitive DEX detection methods.

Usually, methods for the rapid detection of chemical substances are based on biomolecule (e.g., antibodies and aptamer) or artificial molecule (e.g., molecular imprinted polymer) sensors. However, antibody-based assays are expensive and prone to spoilage during storage, and therefore not suitable for use in certain environments. Molecular imprinting polymer sensors require the target to possess high chemical stability and for its structure to have no electrochemical activity. This is due to the physical and chemical properties of DEX; it is difficult to prepare MIPs with high sensitivity and selectivity. In contrast, the aptamer is a short oligonucleotide chain that is selected by systematic evolution of ligands by exponential enrichment (SELEX) in vitro process [13,14]. They can specifically recognize target substances, such as proteins, small molecule drugs, cells, etc. [15,16], in addition to having high specificity, low cost, and good biocompatibility [17]. The production of aptamers is straightforward provided the sequence is known. In addition, their performance is comparable to antigens and antibodies when used as targeting molecules in drug detection and biosensing applications [18].

**Figure 1:**
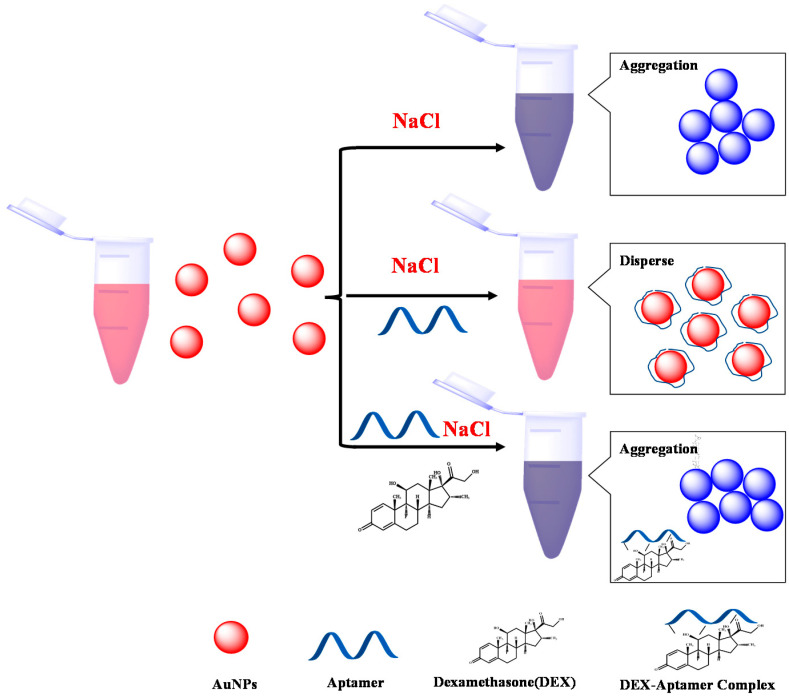
Schematic diagram of the colorimetric method for DEX detection based on aptamers and AuNPs.

In 2019, a high-specific aptamer was developed for electrochemical biosensors used for the detection of DEX in water [19]. However, the interaction mechanism between aptamer and DEX is still unclear. Therefore, in this study, molecular docking simulations and molecular dynamics simulations are used to systematically study the binding sites, binding methods, and spatial configuration changes of aptamers and DEX. Based on this, the colorimetric assay was designed; Figure 1 depicts the principle of the sensing technology we studied. DEX, aptamer, and Au NPs are all part of the sensing system. In the absence of DEX, the aptamer adheres to the surface of Au NPs through electrostatic contact, which keeps the Au NPs dispersed in solution even at high NaCl concentrations. Au NPs that are widely scattered have a red hue. In the presence of DEX, aptamers preferentially form stable complexes with DEX and cannot be adsorbed onto the Au NPs; as a result, the bare Au NPs cannot be stabilized at high NaCl concentrations, and aggregate, resulting in a color change from red to blue. By optimizing each sensing condition and monitoring the variation of the solution color and UV-Vis spectrum, a colorimetric method for rapid detection of DEX was developed. This as-built sensing procedure is efficient, easy to operate, and has a high sensitivity to DEX in milk and glucosamine; the LOD could be reached at 0.5 nmol/mL. This approach has great potential for on-the-spot DEX analysis in food samples.

## 2. Material and Methods

### 2.1. Materials and Reagents

DNA probes (aptamer sequence: 5′-ACA CGA CGA GGG ACG AGG AGT ACT TGC CAA CGA TAA CGT CGT TGG ATC TGT CTG TGC CC-3′) were purchased from Sangon Biotechnology Co., Ltd. (Shanghai, China). HAuCl_4_·H_2_O was purchased from Beijing Chemical Reagent Company (Beijing, China). Dexamethasone was purchased from Shanghai Macklin Biochemical Co., Ltd. (Shanghai, China). Trisodium citrate dihydrate was obtained from Yongsheng Fine Chemicals Company (Tianjin, China). Methanol (CH_3_OH) and Acetonitrile (C_2_H_3_N) were obtained from Fisher Scientific Co., Ltd. (Shanghai, China). Ethyl acetate(C_4_H_8_O_2_) was purchased from Tianjin Beilian Fine Chemicals Development Co., Ltd. (Tianjin, China). Milk and glucosamine samples were obtained at the local market. All experiments were repeated three times, and the standard deviation (error bars) on three sets of measurements were calculated by Origin software.

### 2.2. Apparatus

UV spectra were obtained using a SHIMADZU UV-2700 UV-vis spectrophotometer (SHIMADZU Ltd., Kyoto, Japan), and a JEM-1230 (JEOL Co., Tokyo, Japan) transmission electron microscope (TEM) was used to determine the form and size distribution of Au NPs. A KDC-2044 chilled centrifuge was used for the centrifugation (Zhongke Instrument Co., Hefei, China). The ultrasonic treatment was carried out on a KQ3200DE ultras nicator (Kunshan Instrument Co., Suzhou, China).

### 2.3. Molecular Operating Environment (MOE)-Docking Simulation of Aptamer Bound to DEX

The three-dimensional structure of the Dexamethasone was obtained from the PubChem database (https://pubchem.ncbi.nlm.nih.gov/compound/5743, accessed on 30 November 2021). The energy of the DEX was minimized through Chem3D and converted to mol2 format. The structure of aptamer was predicted by the nucleic acid structure modeling server RNAComposer (https://rnacomposer.cs.put.poznan.pl/, accessed on 4 December 2021) and the molecular operating environment (MOE 2019.1) platform was used to perform geometric optimization and energy minimization of the aptamer. The Lamarckian genetic algorithm was used for molecular docking calculation. The best confirmation of DEX and aptamer was established and the binding energy of the two was computed by optimizing the ideal position and dihedral angle of the DEX molecule. The visualization of the docking results was processed by Pymol 2.1 software.

### 2.4. Molecular Dynamics Simulation

The molecular dynamics simulation process of the aptamer and DEX complex used Desmond 2020 software to hydrogenate the complex, and the water molecule used the simple point charge mode (SPC). A counter ion was added to the system to neutralize and balance the charge of the complex. Desmond’s default setting program was used to achieve system energy minimization and system relaxation process, the Particle Mesh E-wald (PME) method was used to calculate the long-range electrostatic interaction, and the SHAKE bond length limitation algorithm was used to limit all covalent bonds. The molecular dynamics simulation was carried out under the periodic boundary conditions in the normal pressure and temperature (NPT) ensemble. The temperature coupling method was Nose-Hoover. Finally, a 100 ns production MD simulation was performed. After the completion of molecular dynamics, a graphical analysis was performed to monitor the stability of the complex of aptamer and DEX in the solvent.

### 2.5. Preparation of Au NPs

The procedure in the literature for the synthesis of Au NPs by sodium citrate reduction of HAuCl_4_ was used with some modifications [20]. Briefly, 10 mL of sodium citrate solution (38.8 mM) was rapidly injected into a 100 mL boiling solution of HAuCl_4_ (1 mM) under vigorous stirring. Boiling was continued for 10 min while stirring, then the heat was turned off and the solution was stirred for another 10 min. The resulting wine-red solution was cooled to room temperature, then stored in dark glass bottles at 4 °C for further use.

### 2.6. Aptamer Biosensor for Detecting DEX

A typical colorimetric analysis was realized as follows: 300 μL Au NPs and 200 μL 0.07 μM Aptamer were incubated at 25 °C for 30 min. Then, 500 μL of AuNPs-aptamer mixed solution was injected into 200 μL of DEX solutions of different concentrations and reacted at 25 °C for 30 min, followed by adding 200 μL 36.7 mM NaCl and mixing well. The ultimate concentration of DEX was 10, 50, 100, 150, 200, 250, 300, and 350 nmol/mL respectively. Each experiment was performed three times. Afterward, the UV-vis was measured as described above. The spectral data of samples were collected in the wavelength range from 400 to 800 at room temperature.

### 2.7. Selectivity Assay

To test the anti-interference ability of the DEX-sensing system, different analogs of DEX, including prednisone (PDN), hydrocortisone (HC), estradiol(E2), chloramphenicol (CHL), and prepared blank actual sample were added to the sensor system separately, and the difference between the absorbance ratios ΔR(ΔR = R − R_0_) of each sample was monitored and compared. R is absorbance ratio A_650_/A_520_ containing DEX analog, and R_0_ is absorbance ratio A_650_/A_520_ of blank solution. Finally, the results were analyzed for significance.

### 2.8. Application in Milk and Glucosamine Samples

2 g milk and 100 mg glucosamine were added to 30 mL ethyl acetate, and acetonitrile, respectively. The solution was vortexed for 30 s and sonicated for 10 min. The mixtures were then centrifuged at 4500× *g* rpm for 15 min, and the supernatant was separated and transferred into hydrophile lipophile balance (HLB) extraction cartridges. The filtrate was collected and evaporated to dryness under a nitrogen stream in a water bath (40 °C). The residue was dissolved in 30 mL of methanol-H_2_O solution (3:7, *v*/*v*).

## 3. Results and Discussion

### 3.1. MOE-Docking and MD Simulation Results

**Figure 2:**
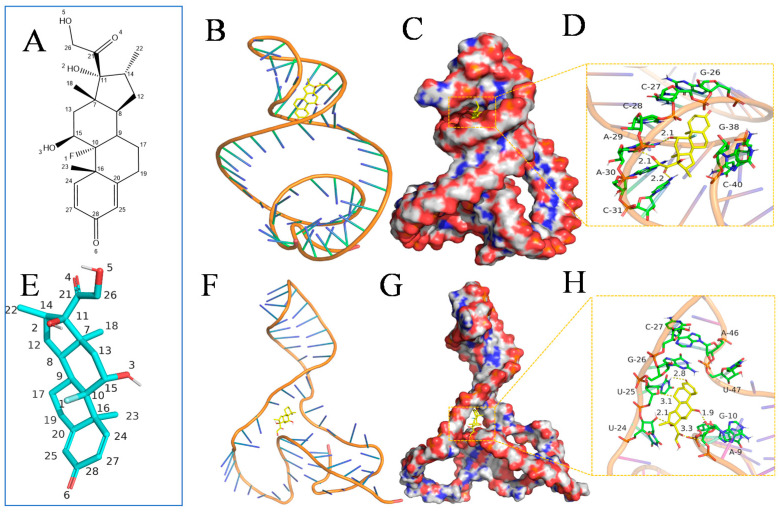
The atomic number of the DEX (**A**,**E**). The binding mode of the aptamer with DEX after MOE-Docking (**B**). The 3D structure of the complex (**C**). The electrostatic surface of the aptamer (**D**). The binding mode of the aptamer with DEX after MD (**F**). The 3D structure of the complex (**G**). The electrostatic surface of the aptamer, showing the detail binding mode of aptamer with DEX. The yellow dash represents the hydrogen bond (**H**).

To explore the specific binding mode of aptamer with DEX molecules, we used molecular docking technology to predict and assess the binding sites of aptamer with DEX molecules. The modeling results demonstrate that the interactions of DEX molecule with base sites of G-26, C-27, C-28, A-29, A-30, C-31, G-38, and C-40 on aptamer were mainly through hydrophobic interaction and hydrogen bonds. Of these, the core structure of DEX is composed of multiple six-membered carbon rings and has strong hydrophobicity. It has a certain hydrophobic interaction with the pyridine ring and nitrogen heterocycle of the site base, In addition, the No. 3 hydroxyl group, No. 5 hydroxyl group, and No. 4 carbonyl groups on DEX molecules form hydrogen bonds with the bases A-29, A-30, and C-31, with bond lengths of 2.1 Å, 2.1 Å, and 2.2 Å, respectively. These hydrogen bonds are relatively short and are much shorter than 3.5 Å in length. They bind strongly to the aptamer site and have an important contribution to the formation of stable complexes and the specific recognition of DEX. Combining Figure 2B–D, DEX is wrapped in the cavity formed by aptamer and matched well. It shows that DEX can form a stable complex with the aptamer. The DEX molecule has a binding energy of 5.64 kcal/mol with the aptamer. Additional structural investigations of aptamers could be based on the above findings.

To further study the interaction between DEX molecules and aptamer, we performed a 100 ns molecular dynamics simulation on the complex. It was found that the binding site of DEX and aptamer had changed, and the bases that interacted with it mainly include G-26, C-27, U-25, U-24, G-10, A-9, U-47, and A-46. Combining Figure 2F–H, we found that the aptamer conformation had also undergone major changes. The entire aptamer began to regularly form a helical conformation, and hydrogen bonds began to form between base pairs, which made the conformation more stable. In addition, the position of DEX had moved to a certain extent, but it could still be linked to multiple bases (No. 6 carbonyl group and U-25, No. 3 hydroxyl group and U-24, No. 4 carbonyl group and G-10, No. 3 hydroxyl and A-9) forming a stable hydrogen bond interaction, which effectively anchors DEX to the site. At the same time, DEX can always be combined in the pocket formed by the aptamer during the entire process, without separation, forming a stable complex.

In addition, the Root-Mean-Square Deviation (RMSD) of the aptamer increased with simulation time and then plateaued after 30 ns. The average RMSD was 3.4 Å in the binding pocket (Figure S1), reflecting the excellent stability of the composite material; there was no off-target phenomenon in the simulated environment. This further shows that DEX can be stably anchored at the active site of the aptamer. At the same time, 30 ns to reach equilibrium indicates that the DEX and aptamer cavity match well, and the conformation can be adjusted quickly to reach a dynamic equilibrium state.

### 3.2. The Operation Principle of the Developed Aptasensor

**Figure 3:**
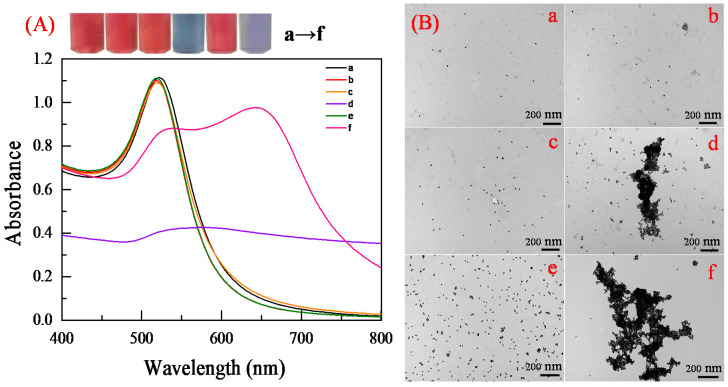
The UV absorption spectra and photograph (**A**) of 300 μL Au NPs dispersed in the sensing system: as-prepared (**a**), in the presence of 0.07 μM aptamer (**b**), in the presence of 350 nmol/mL DEX (**c**), in the presence of 36.7 mM NaCl (**d**), in the presence of 36.7 mM NaCl and 0.07 μM DEX-aptamer (**e**), and in the presence of 36.7 mM NaCl, 0.07 μM aptamer, and DEX at concentrations of 350 nmol/mL (**f**), shown in their respective transmission electron microscopy images (**B**).

An aqueous solution containing Au NPs can be well dispersed and stabilized with the electrostatic repulsion generated by citrate root; the solution is bright red, which is consistent with the TEM image of Au NPs (Figure 3Ba). The average diameter of the as-prepared Au NPs is 13 nm (Figure S2). The absorption spectrum of Au NPs shows an absorption peak at 520 nm (Figure 3A). The molar extinction coefficient of these AuNPs is 2.7 × 10^8^ cm^−1^M^−1^ at 520 nm [21,22], which indicates that the molar concentration is approximately 4.04 × 10^−9^ mol L^−1^ by Lambert Beer’s law. NaCl, Aptamer, and DEX were mixed with Au NPs, respectively. The AuNPs were not induced to aggregation by aptamers or DEX; there was no variation in dispersion state and color (Figure 3b,c). In the presence of NaCl, a high concentration of cations broke the electrostatic equilibrium of AuNPs solution, demonstrating NaCl-induced aggregation of AuNPs; the solution color changed from red to blue. The absorbance of these AuNPs decreased at 520 nm (A_520_) and increased at 650 nm (A_650_) (Figure 3d). In the presence of aptamer and NaCl, the aptamer could counteract the NaCl-induced aggregation, resulting in a decrease in the absorbance of AuNPs at 650 nm (A_650_) and a significant increase at 520 nm (A_520_); the monodisperse state was shown by a red color (Figure 3e). When DEX was introduced to the solution, DEX was preferentially bound specifically to the aptamer and formed stable complexes. Then, the NaCl induced the aggregation of Au NPs (Figure 3f), and the color of the solution changed from wine red to blue. The result indicated that the AuNPs could be used as colorimetric probes for detecting DEX.

### 3.3. Optimization of Experimental Conditions

The proposed DEX analysis is based on the dispersed to aggregate state change of AuNPs caused by the analyte. As a result, assay settings such as NaCl concentration, aptamer concentration, interaction time, and so on have a significant impact on the performance of the established DEX assay.

The ability of Au NPs to remain dispersed in an aqueous solution is mostly due to electrostatic stabilization. We employed the absorbance ratio to determine the degree of aggregation of the Au NPs in this assay, which has been demonstrated to be quite accurate in prior investigations [23] because NaCl was employed to control the degree of aggregation of the Au NPs. To assess the efficiency of aggregation, several concentrations of NaCl were added to Au NPs solutions, ranging from 10.0 to 43.3 mM, and the A_650_/A_520_ ratio was measured. The color of the Au NPs solutions gradually changed from wine red to purple to blue (Figure S3). The absorbance of Au NPs at 520 nm gradually reduced as the NaCl concentration increased, which was also accompanied by an increase in absorbance at 650 nm. The absorption ratio (A_650_/A_520_) rapidly increased from 10 to 36.7 mM before plateauing at 36.7 mM, indicating that AuNP aggregation was nearly complete at a concentration of 36.6 mM (Figure S3b).

The effects of the aptamer concentration on the absorbance ratio were explored since, in addition to NaCl concentration, the aptamer concentration is a significant factor that impacts the analytical performance of the assay for DEX detection. The absorbance ratio declined with increasing aptamer concentration from 0.01 to 0.2 μM, reaching a plateau at 0.07 μM (Figure S4). Consequently, the ideal aptamer concentration was determined to be 0.07 μM.

In addition, the incubation time of DEX with aptamer, and the reaction time of AuNPs and aptamer, were also investigated as key parameters for colorimetric assays. First, the effect of reaction time on the absorbance ratio (Figure S5a) was studied and the A_650_/A_520_ was accompanied by an increase from 0 to 30 min before plateauing at 30 min, indicating the aptamer takes 30 min to wrap on the surface of Au NPs. Next, the effect of incubation time on absorbance ratio was studied after determining the reaction time as 30 min; the A_650_/A_520_ had no significant variation (Figure S5b), which demonstrates the fast dynamics between the DEX and aptamer. However, to attain the ideal analytical performance for this colorimetric method, the optimal incubation time and reaction time for subsequent studies were determined to be 30 min.

### 3.4. Quantitative Determination of DEX

**Figure 4:**
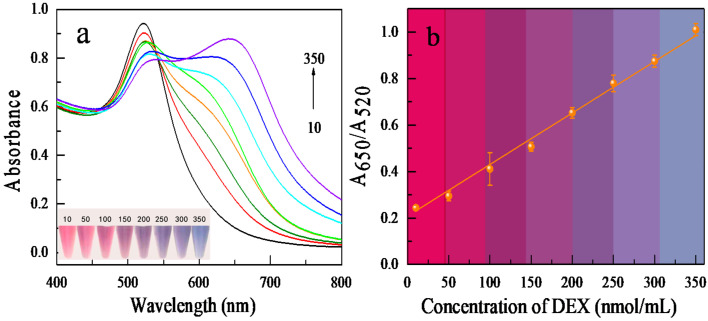
The aptamer-based DEX detection assay’s sensitivity. (**a**) Color and UV absorption spectra of sensing systems treated with 10, 50, 100, 150, 200, 250, 300, and 350 nmol/mL DEX. (**b**) A plot depicting the linear response at different concentrations.

DEX solutions with concentrations ranging from 10 to 350 nmol/mL were detected under the parameters of optimal NaCl concentration (36.7 mM), aptamer concentration (0.07 μM), and assay protocol time (30 min) to quantitatively assess the biosensor’s sensitivity. As illustrated in Figure 4, the UV-visible absorption spectra and color variations of AuNPs were monitored as a function of DEX concentration. The absorbance of the AuNPs colloidal solution at 520 nm (A_520_) reduced with DEX concentrations ranging from 10 to 350 nmol/mL, but it rose at 650 nm (A_650_). In addition, the solution’s coloration changed from red to blue. When the DEX concentration was greater than 50 nmol/mL, the color alteration of the solution could be observed, and the method’s visual limit was reached at 50 nmol/mL. Figure 4b depicts the dynamic change in the A_650_/A_520_ ratio after DEX was added. The absorbance ratio (A_650_/A_520_) had an excellent linear relationship with DEX concentrations in the range of 10 to 350 nmol/mL (linear equation: y = −0.002x + 0.209, correlation coefficient r^2^ = 0.997). The detection limit (LOD, 3σ/S) was determined to be 0.5 nmol/mL. At the same time, we have established a visual colorimetric interval to facilitate the evaluation of DEX concentration with the naked eye without instruments (various colors of solution in the presence of 10–350 nmol/mL of DEX, with rose-red = 10–50, Pink = 50–100, plum = 50–100, light purple = 150–200, purple = 200–250, indigo = 250–300, Grey blue = 300–350).

### 3.5. Selectivity of the Assay for DEX Detection

DEX analogs and some substances in milk or glucosamine samples that may be extracted by organic solvents may interfere with the stability of Au NPs and the specificity of aptamers. Therefore, it is vital to investigate the impact of interfering substances on biosensors for the detection of DEX. As shown in Figure S6A, we found that the sample extract solution did not cause Au NPs to aggregate, and did not interfere with the aptamer’s anti-aggregation effect on Au NPs. We also studied the influence of some DEX analogs such as E2, HC, PDN, and CHL. The ΔR was calculated; the results are shown in Figure S6B. And one-way analysis of variance (ANOVA) was used to analyze the significance using the DEX group and DEX analogs separately; DEX as a control group is significant (*p* < 0.01), which demonstrated that only DEX can bind with aptamer and cause the aggregation degree of AuNPs induced by NaCl. According to all the data, it can be seen that our sensor has excellent selectivity.

### 3.6. Detection of DEX in Milk and Glucosamine Samples

A series of studies were performed using milk and glucosamine with three different doses of DEX (100, 200, and 300 nmol/mL) to evaluate the performance of the designed aptasensor to detect DEX in real samples. After adding the prepared sample to the colorimetric sensor, the concentration of DEX was evaluated with the naked eye by observing the color change of AuNPs with the previously established visual colorimetric interval. This can be easily seen by the naked eye when the DEX concentration is higher than 50 nmol/mL, and the UV spectrophotometer is also used to record the value of A650/A520 to further calculate the concentration of DEX. The results for each sample are listed in Table 1. The mean sample recoveries ranged from 93.6% to 117%. Compared with existing detection methods (see Table 2), the designed aptasensor performs excellently. Together, the above results demonstrated the feasibility of a DEX-based colorimetric sensor for ultra-high sensitivity, fast and convenient operation, low cost, and good anti-matrix interference capability. These properties of the aptasensor also make it a broad and versatile detection technique with great potential for rapid DEX detection in food.

## 4. Conclusions

In summary, a sensitive and selective colorimetric detection method based on aptamers and AuNPs was successfully developed to detect DEX. Following sensing conditions optimization, DEX could be detected in the range of 10–350 nmol/mL, with a DEX detection limit of 0.5 nmol/mL. When the DEX concentration reached as high as 50 nmol/mL, it could be detected by naked eyes. The detection performance of this method can satisfy the MRLs of DEX in food. By adopting aptamer as the recognition element, the sensor displays a good selectivity. The molecular docking and molecular dynamics simulations studies have shown that the detection performance of the colorimetric sensor mainly depends on the affinity of the aptamer with DEX. The characteristics of aptamers such as good stability, easy modification, and low cost make the truncation optimization strategy of aptamers the focus of future research. The mechanistic studies provide a theoretical basis for the truncation and optimization of aptamers.

## Figures and Tables

**Table 1:** Spike recovery results for DEX.

Sample	Added (nmol/mL)	Recovery
glucosamine	100	(104 ± 3.2)%
	200	(109.6 ± 2.5)%
	300	(100.7 ± 5.1)%
milk	100	(114 ± 4.8)%
	200	(93.6 ± 4.8)%
	300	(117 ± 2.9)%

**Table 2:** Existing Dexamethasone Determination Method Comparison Chart.

Method	Linear Range	LOD	Applications	Recovery	Ref.
Electrochemical aptasensor	2.5–100 nM	2.12 nM	tab water and drinking water	81.5–103.2%	[19]
Immunochromatography	–	Milk: 0.3 ng/mL pork: 0.7 μg/kg	milk and pork	80.0–122.8%	[24]
Electrochemical aptasensor	10–500 μg/mL	3.59 μg/mL	herbal medicine samples	-	[25]
HPLC	–	water = 6 ng/mL Feed = 190 ng/g	water and feed for meat-producing animals	99.4 ± 1.3%	[26]
Electrochemical sensor	0.05 to 30 mM	3.0 nM	human urine and serum samples	97.0–102.0%	[27]
HPLC	–	10 ng/mL	human plasma	96.96–106.07%	[28]
Hanging mercury drop electrode	25.5–122.3 µM	7.6 µM	drug sample	99.8–100%	[29]
Square-wave adsorptive voltammetry	0.0498–0.61 µM	2.54 nM	eye drops, injectable, elixir	94.14–112.41%	[30]
Colorimetric biosensor	0.1–9 ng/mL	2.0 μg/kg	food supplements and cosmetic samples	–	[31]
Colorimetric biosensor	10–350 nmol/mL	0.5 nmol/mL	milk and glucosamine	93.6–117%	this assay

## Data Availability

All data generated or analyzed during this study are included in this published article (and its Supplementary Information Files).

## Supplementary Materials

The following supporting information can be downloaded at: https://www.mdpi.com/article/10.3390/bios12040242/s1, Figure S1. The RMSD plot during molecular dynamics simulations of aptamer with DEX; Figure S2. Size distribution of 100 Au NPs with an average diameter of 13 nm; Figure S3. Effect of NaCl concentration on spectra(a) and absorbance ratio(b) of the AuNPs in the sensing system. The AuNPs volume was 300 μL; Figure S4. In the absence of DEX, the effect of aptamer concentration on spectra (a) and absorbance ratio (b). The AuNPs volume was 300 μL and the NaCl concentration was 36.7mM; Figure S5. Changes in absorbance ratio as a function of reaction time (a). The AuNPs volume was 300 L, the NaCl concentration was 36.7 mM, and the aptamer concentration was 0.07 μM. When the fixed reaction time is 30 min, the absorbance ratio changes with the incubation time (b). The volume of 36.7 mM NaCl, 0.07 μM aptamer, and DEX at concentrations of 350 nM; Figure S6. (A): Absorption spectra of the effect of the extract of real sample on the Au NPs. (a) 300 µL Au NPs + 200µL H2O + 200 µL aptamer+200 µL NaCl; (b) 300 µL Au NPs + 200µL extract solution of glucosamine + 200 µL aptamer+200 µL NaCl; (c) 300 µL Au NPs + 200µL extract solution of milk + 200 µL aptamer+200 µL NaCl. (B): The cross reactivity of the DEX aptasensor against 30 μg ml^−1^ of DEX, HC, PDN, E2 and CHL.
