# Supplementary material for: Based on Unmodified Aptamer-Gold Nanoparticles Colorimetric Detection of Dexamethasone in Food
Source: Biosensors (Basel). 2022 Apr 14;12(4):242. doi: 10.3390/bios12040242 (PMC9029452; doi:10.3390/bios12040242)
Supplement: Supplementary file 1 [file biosensors-12-00242-s001.zip › biosensors-1661469-supplementary.pdf]

Supporting Information

Based on Unmodified Aptamer-Gold Nanoparticles Colorimetric Detection of Dexamethasone in Food

Yadi Qin [1], Hayilati Bubiajiaer [1], Jun Yao [1,*] and Minwei Zhang [2,*]

[1] School of Pharmacy, Xinjiang Medical University, Xinyi Road, Urumqi 830054, China; xydqyd@stu.xjmu.edu.cn (Y.Q.); 107602201301@stu.xjmu.edu.cn (H.B.)
[2] College Life Science & Technology, Xinjiang University, Shengli Road, Urumqi 830046, China
* Correspondence: xydyaojun2022@xjmu.edu.cn (J.Y.); zhangmw@xju.edu.cn (M.Z.); Tel.: +86-18999250641 (J.Y.); +86-13999258239 (M.Z.)

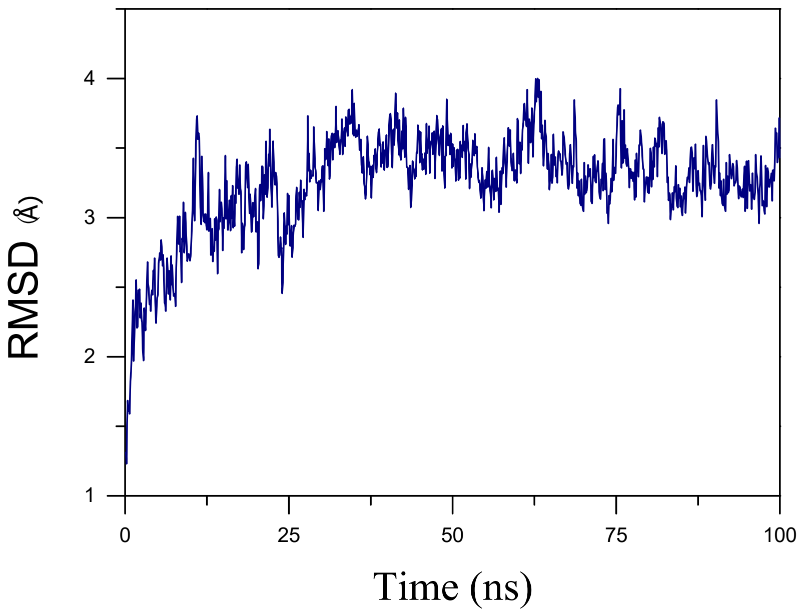

Figure S1. The RMSD plot during molecular dynamics simulations of aptamer with DEX.

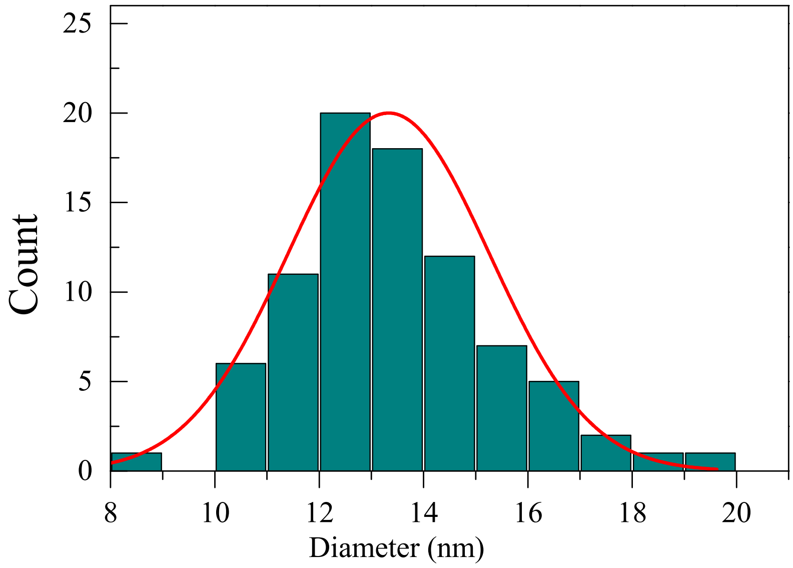

Figure S2. Size distribution of 100 Au NPs with an average diameter of 13nm.

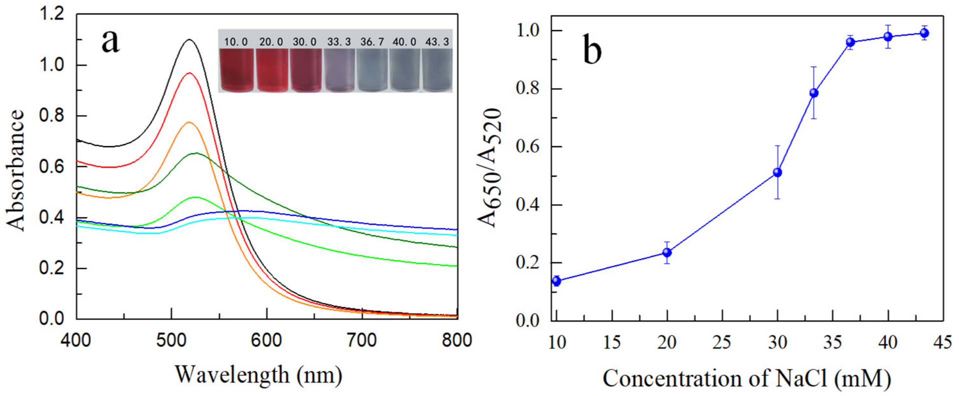

Figure S3. Effect of NaCl concentration on spectra (**a**) and absorbance ratio (**b**) of the AuNPs in the sensing system. The AuNPs volume was 300 μL.

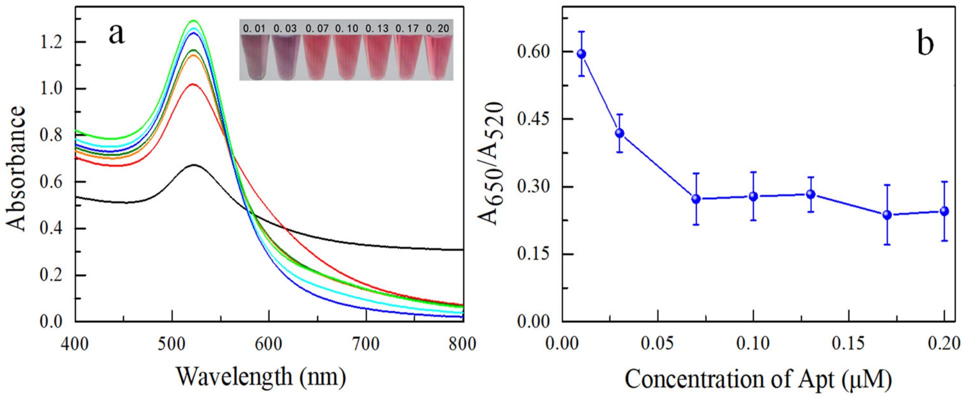

Figure S4. In the absence of DEX, the effect of aptamer concentration on spectra (**a**) and absorbance ratio (**b**). The AuNPs volume was 300 μL and the NaCl concentration was 36.7 mM.

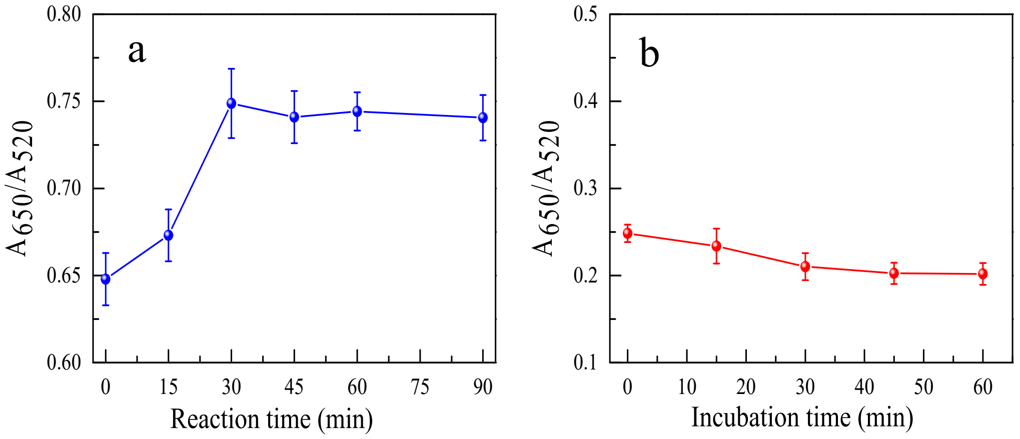

Figure S5. Changes in absorbance ratio as a function of reaction time (**a**). The AuNPs volume was 300 L, the NaCl concentration was 36.7 mM, and the aptamer concentration was 0.07 μM. When the fixed reaction time is 30 min, the absorbance ratio changes with the incubation time (**b**). The volume of 36.7 mM NaCl, 0.07 μM aptamer, and DEX at concentrations of 350 nM.

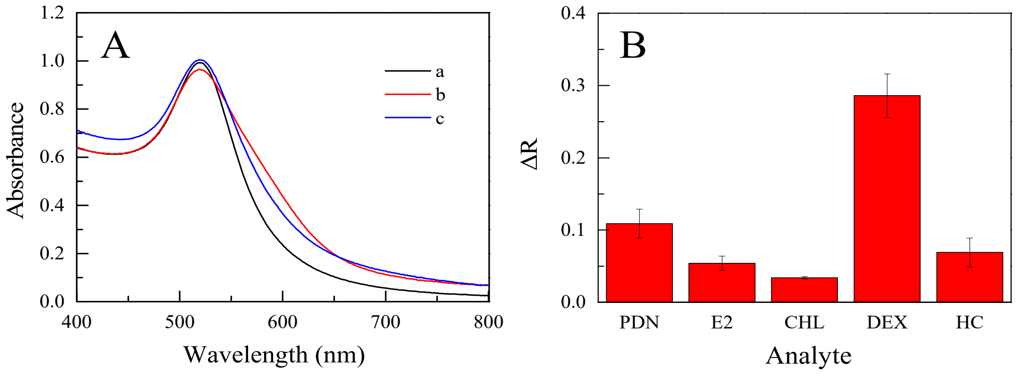

Figure S6. (**A**): Absorption spectra of the effect of the extract of real sample on the Au NPs. (a) 300 μL Au NPs + 200 μL H2O + 200 μL aptamer+200 μL NaCl; (b) 300 μL Au NPs + 200 μL extract solution of glucosamine + 200 μL aptamer+200 μL NaCl; (c) 300 μL Au NPs + 200 μL extract solution of milk + 200 μL aptamer+200 μL NaCl. (**B**):The cross reactivity of the DEX aptasensor against 30 μg ml−1of DEX, HC, PDN, E2 and CHL.
